# Temporal trends and correlates of *Helicobacter pylori* prevalence in children undergoing upper gastrointestinal endoscopy

**DOI:** 10.1007/s00431-026-06908-4

**Published:** 2026-04-09

**Authors:** Kadir Işık, Burcu Volkan, Deniz Ertem

**Affiliations:** 1 Department of Pediatrics, Marmara University Faculty of Medicine, İstanbul, Turkey; 2 Division of Pediatric Gastroenterology, Hepatology and Nutrition, Marmara University Faculty of Medicine, İstanbul, Turkey

**Keywords:** Prevalence, Upper gastrointestinal endoscopy, Dyspepsia, Growth impairment, Iron deficiency anemia

## Abstract

This study aims to evaluate temporal trends in *Helicobacter pylori* prevalence in symptomatic children undergoing upper gastrointestinal endoscopy and to identify demographic, clinical, endoscopic, and histopathological factors associated with *H. pylori* infection. We retrospectively evaluated children aged 0–18 years who underwent upper gastrointestinal endoscopy for various gastrointestinal symptoms with histopathological examination and rapid urease testing between 2010 and 2020. Patients with concordant diagnostic results were included. Demographic characteristics, symptoms, endoscopic and histopathological features were analyzed. Temporal trends in *H. pylori* prevalence were assessed, and factors associated with *H. pylori* were evaluated using multivariate logistic regression analyses. Among 1300 eligible patients, 335 (25.8%) were *H. pylori*–positive. The prevalence of *H. pylori* declined significantly over the study period, decreasing from 47.9% in 2010 to 23.4% in 2020. *H. pylori* positivity was associated with older age and lower height Z-score. Dyspepsia and refractory iron deficiency anemia, endoscopically antral nodularity, pangastritis, and duodenal ulcer were more frequent in *H. pylori*–positive patients and remained independently associated with *H. pylori* infection. Although *H. pylori* infection was more frequent in patients with glandular atrophy and intestinal metaplasia, no independent association was found between the lesions and *H. pylori* after multivariate adjustment.

*Conclusion*: In symptomatic children who underwent endoscopy, a nearly 50% decline in *H. pylori* prevalence was observed over a 10-year period. The infection was associated with impaired linear growth, refractory iron deficiency anemia, dyspeptic symptoms, and characteristic endoscopic findings; this underlines the continued clinical importance of *H. pylori* infection in pediatric practice.

**Table:** 

**What is Known:**	
• *Helicobacter pylori infection is commonly acquired in childhood.* • *The decline in H. pylori prevalence in children is less consistent compared to adults, and its clinical associations remain controversial.*	
**What is New:**	
• *We observed a nearly 50% decline in H. pylori prevalence over a 10-year period, from 47.9% in 2010 to 23.4% in 2020 among symptomatic children, with the most pronounced decline in school-aged children and adolescents.* • *In this cohort, precancerous lesions were uncommon (gastric atrophy 2.3%, intestinal metaplasia 1.38%) and, although H. pylori infection was nearly twofold higher in children with these lesions, they were not independently associated with H. pylori infection.*

• *Helicobacter pylori infection is commonly acquired in childhood.*

• *The decline in H. pylori prevalence in children is less consistent compared to adults, and its clinical associations remain controversial.*

• *We observed a nearly 50% decline in H. pylori prevalence over a 10-year period, from 47.9% in 2010 to 23.4% in 2020 among symptomatic children, with the most pronounced decline in school-aged children and adolescents.*

• *In this cohort, precancerous lesions were uncommon (gastric atrophy 2.3%, intestinal metaplasia 1.38%) and, although H. pylori infection was nearly twofold higher in children with these lesions, they were not independently associated with H. pylori infection.*

## Introduction

*Helicobacter pylori* (*H. pylori*) infection is one of the most prevalent bacterial infections globally, and chronic *H. pylori* infection is associated with the development of peptic ulcer disease, chronic gastritis, gastric cancer, and mucosa-associated lymphoid tissue lymphoma [1]. It is mostly acquired in childhood and continues throughout life if left untreated [2]. Clinically, *H. pylori* infection often presents with dyspeptic symptoms in adults [3]. Recent pediatric studies report no correlation between *H. pylori* infection and gastrointestinal symptoms among children undergoing endoscopy for dyspeptic complaints [4, 5].

The global prevalence of *H. pylori* infection has declined in the last two decades [6, 7], but 45–50% of the adult population is still infected with *H. pylori* [6–8]. This decline is attributed to successful eradication of *H. pylori* infection in adults, improved living conditions, better hygiene, and sanitation [9].

Recent pediatric data indicate that *H. pylori* prevalence varies significantly according to socioeconomic status, with rates around 15–20% in high–income areas and approaching 40–50% in low- and middle-income areas [6, 10]. While a decreasing trend in pediatric *H. pylori* prevalence has been observed over time, this reduction is less pronounced and less consistent than that reported in adults [10]. Considerable variability in published prevalence estimates likely reflects several factors: regional differences in study settings, heterogeneity in diagnostic methods, differences in sampled populations, and variation in the age ranges of study participants.

The present study aimed to assess temporal changes in the prevalence of *H. pylori* infection over a 10-year period in children undergoing upper gastrointestinal endoscopy for various gastrointestinal symptoms, and to evaluate the demographic characteristics, presenting symptoms, and endoscopic and histopathological findings associated with *H. pylori*.

## Patients and methods

### Diagnosis of *H. pylori* infection

Medical records of children who underwent upper gastrointestinal endoscopy (UGE) for gastrointestinal complaints in the Pediatric Gastroenterology outpatient clinic between 2010 and 2020 were retrospectively reviewed. Endoscopy and pathology reports were retrieved from a prospectively recorded institutional database. This symptom-based cohort included children undergoing their first UGE, irrespective of the final diagnosis. Patients who had previously received *H. pylori* eradication therapy or had primary immunodeficiency or chronic systemic disease requiring immunosuppressive treatment were excluded. Conditions such as inflammatory bowel disease, celiac disease, or eosinophilic esophagitis were not applied as a priori exclusion criteria, as these diagnoses were often established after endoscopic and histological evaluation. To ensure standardized assessment of *H. pylori* infection, only procedures including both antral and corpus biopsies with rapid urease testing (RUT) were considered eligible. *H. pylori* diagnosis was based on histopathology combined with a positive RUT in accordance with the 2016 European Society for Paediatric Gastroenterology, Hepatology and Nutrition (ESPGHAN) and North American Society for Pediatric Gastroenterology, Hepatology and Nutrition (NASPGHAN) guidelines [11]. Patients were considered negative if both histopathology and RUT were negative, and those with discordant results were excluded to ensure diagnostic consistency.

### Demographic and anthropometric assessment

Data on age, gender, height, weight, and date of endoscopy were extracted from hospital records. Patients were grouped into 3 age groups: preschool (0–5 years), school-age (5–12 years), and adolescent (12–18 years). Z-scores for height, weight, and body mass index (BMI) were calculated using published national reference values based on anthropometric measurements obtained at the time of endoscopy [12]. Growth failure was defined as height-for-age and/or weight-for-age Z-score below − 2 SD, or weight-for-length Z-score below − 2 SD in children younger than 2 years, or BMI-for-age Z-score below − 2 SD in children aged ≥ 2 years. Growth faltering was defined as a downward crossing of ≥ 2 major percentile lines on growth charts when longitudinal data were available. Children meeting criteria for either growth failure or growth faltering were analyzed within the same clinical category.

### Clinical assessment

The patients’ files were reviewed to determine presenting symptoms for undergoing UGE, including dyspeptic symptoms, reflux-related complaints, hematemesis and/or melena, growth faltering/failure, refractory iron deficiency anemia (IDA), and other causes. Dyspeptic symptoms were defined as epigastric pain or burning, postprandial fullness, early satiety, and nausea. Reflux-related symptoms included regurgitation, waterbrash, retrosternal discomfort, and heartburn. Anemia was defined as hemoglobin values below − 2 SD for age and gender [13]. Refractory IDA was defined as persistent anemia despite appropriate oral iron supplementation for at least 3 months, according to medical records.

### Endoscopic and histopathological assessment

Endoscopy reports of the patients were evaluated for macroscopic findings, including mucosal erythema, ulceration, and nodularity in the esophagus, corpus, antrum, and duodenum. RUT results were also recorded.

Pathology reports of the patients were reviewed for the histopathological presence of *H. pylori* infection. The Updated Sydney System was used to classify gastritis [14], and the presence of chronic inflammation, active inflammation, gastric atrophy, intestinal metaplasia, and *H. pylori* density were evaluated, and each was scored on a scale from 0 to 3 (0: normal, 1: mild, 2: moderate, 3: marked changes).

### Statistical analysis

Statistical analyses were performed using the Statistical Package for the Social Sciences (SPSS), version 20. Continuous variables were summarized as mean ± SD or median with interquartile range (IQR) and minimum–maximum (min–max) values, as appropriate. Categorical variables were expressed as numbers (*n*) and percentages (%).

Associations between categorical variables were assessed using the Pearson chi-square test, Yates’ correction, Fisher’s exact test, or the linear-by-linear association test, as appropriate. Comparisons between independent groups were performed using the independent-samples *t*-test or the Mann–Whitney *U* test according to data distribution. Trends in *H. pylori* prevalence over time were evaluated using the Cochran–Armitage trend test, and annual prevalence rates were presented with 95% confidence intervals (CIs).

Factors associated with *Helicobacter pylori* positivity were examined using multivariate binary logistic regression analyses. Given the heterogeneity and potential collinearity among variables representing different clinical and biological domains, separate regression models were constructed for predefined variable groups. The first model included clinical and demographic variables (age, sex, height Z-score, dyspepsia, and refractory IDA). The second model evaluated endoscopic findings. The third model assessed histopathological variables. All variables were entered simultaneously using the enter method. Results were reported as odds ratios (ORs) with 95% CIs. Statistical significance was defined as *p* < 0.05.

## Results

### Prevalence and temporal trends of *H. pylori* infection

A total of 1432 pediatric patients who underwent both histopathological examination and RUT for *Helicobacter pylori* infection between 2010 and 2020 were included in the study. Of these, 132 patients were excluded due to discordant results between the diagnostic methods. Among the remaining 1300 patients, 335 (25.8%) tested positive for *H. pylori* infection.

**Fig. 1:**
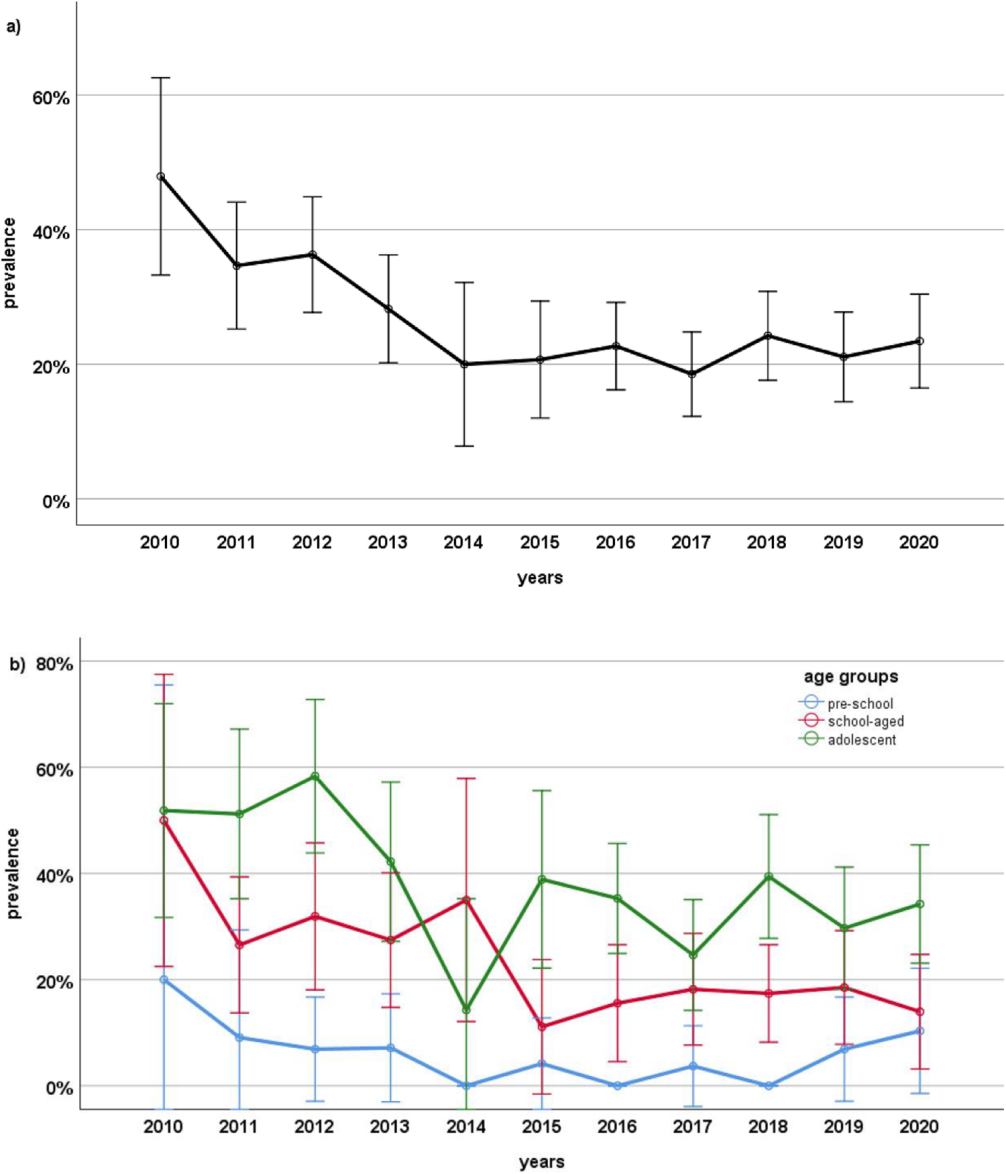
Annual change in *H. pylori* prevalence among all children undergoing endoscopy (**a**) and change in *H. pylori* prevalence by age groups (**b**)

When analyzed by year, *H. pylori* prevalence among pediatric patients was 47.9% (23/48) in 2010, 34.7% (35/101) in 2011, 36.3% (45/124) in 2012, 28.2% (35/124) in 2013, 20.0% (9/45) in 2014, 20.7% (18/87) in 2015, 22.7% (37/163) in 2016, 18.5% (28/151) in 2017, 24.2% (40/165) in 2018, 21.1% (31/147) in 2019, and 23.4% (34/145) in 2020. A significant decline in prevalence was observed during the 2010–2020 period (*p* < 0.001) (Fig. 1a). Age-stratified analysis of annual prevalence showed a decline in preschool children from 20.0% (1/5) to 10.3% (3/29), in school-aged children from 50.0% (8/16) to 14.0% (6/43), and in adolescents from 51.9% (14/27) to 34.2% (25/73). The decline was statistically significant in school-aged children and adolescents (*p* < 0.001 for both), but not in preschool children (*p* = 0.67) (Fig. 1b).

### Patient characteristics, indications, and symptoms

The demographic and anthropometric characteristics of the patients and their *H. pylori* status were shown in Table 1. The infection rate was found to be 27% in 700 female and 24.3% in 600 male patients, with no significant difference between genders. The median age was significantly higher in the *H. pylori*-positive patients, and the prevalence increased significantly across age groups. Height and weight Z-scores were significantly lower in the *H. pylori*–positive compared with the *H. pylori*–negative group, whereas BMI Z-scores were similar between the two groups (Table 1).

**Table 1:** Comparison of demographic and anthropometric characteristics of *H. pylori*–positive and *H. pylori*–negative patients

Demographic and anthropometric data		*H. pylori* (−) (*n* = 965)	*H. pylori* (+) (*n* = 335)	*p*
Median age (IQR) (min–max)		9.7 (8.9) (0.1–18)	14 (5.3) (1.8–18)	< 0.001*
Age groups	Pre-school *n* = 251	238 (94.8%)	13 (5.2%)	< 0.001**
	School-aged *n* = 476	371 (77.9%)	105 (22.1%)	
	Adolescent *n* = 573	356 (62.1%)	217 (37.9%)	
Gender	Female *n* = 700	511 (73%)	189 (27%)	0.273**
	Male *n* = 600	454 (75.7%)	146 (24.3%)	
Family history of GIS complaints	Negative *n* = 668	506 (75.7%)	162 (24.3%)	0.184**
	Positive *n* = 476	344 (72.3%)	132 (27.7%)	
Weight Z-score mean (± SD) (min–max)		− 0.82 (± 1.57) (−6.82–4.52)	− 1.05 (± 1.6) (− 7.52–2.77)	0.020***
Height Z-score mean (± SD) (min–max)		− 0.73 (± 1.45) (− 6.97–4.68)	− 0.98 (± 1.57) (− 6.81–4.32)	0.008***
BMI Z-score mean (± SD) (min–max)		− 0.53 (± 1.51) (− 6.47–4.50)	− 0.60 (± 1.46) (− 5.44–2.84)	0.469***

*Mann–Whitney *U* test

**Pearson chi-square test

***Independent samples *T*-test

The most common indication for endoscopy among the evaluated patients was dyspepsia (51.1%) (Table 2), and *H. pylori* prevalence was significantly higher in patients with dyspeptic symptoms in univariate analysis. Of the 371 patients identified as anemic, 97 (26.1%) were *H. pylori*-positive, while of the 929 non-anemic patients, 238 (25.6%) were *H. pylori-*positive and there was no significant difference between the two groups (*p* = 0.85). Yet, *H. pylori* prevalence was significantly higher among patients who underwent endoscopy due to refractory IDA (Table 2).

**Table 2:** Association of presenting symptoms with *H. pylori* infection

Patients’ symptoms (*n*)	*H. pylori* (+)	p☨
Dyspepsia (*n* = 665)	211 (31.7%)	< 0.001*
Reflux symptoms** (***n* = 291)	86 (29.6%)	0.094*
Hematemesis and/or melena (*n* = 202)	55 (27.2%)	0.606*
Refractory anemia (*n* = 81)	32 (39.5%)	0.005**
Growth faltering/failure (*n* = 276)	67 (24.3%)	0.523*

*Pearson chi-square test

**Yates’ correction test

☨*p* values were calculated by comparing *H. pylori* positivity between patients with and without the respective symptom

### Endoscopic and histopathological findings

Among *H. pylori*-positive patients, antral erythema was observed more frequently than corpus erythema, and most patients with corpus involvement also had antral changes, consistent with pangastritis. Antral nodularity was the most frequent endoscopic finding in the *H. pylori*–infected group. Accordingly, *H. pylori* prevalence was significantly higher in patients with antral nodularity, and in those with endoscopic pangastritis. In contrast, *H. pylori* infection was significantly less frequent in patients with antral gastritis without nodularity. Furthermore, *H. pylori* infection was significantly associated with duodenal ulcers but no such association was observed with gastric ulcers (Table 3). Notably, *H. pylori* infection was detected in 53 of 737 patients (7.2%) with endoscopically normal gastric mucosa.

**Table 3:** Association of endoscopic findings with *H. pylori* infection

Endoscopic findings (*n*)	*H. pylori* (+)	*p*☨
Esophagitis (*n* = 151)	44 (29.1%)	0.314*
Antral gastritis without nodularity (*n* = 161)	20 (12.4%)	< 0.001*
Antral nodularity (*n* = 320)	252 (78.8%)	< 0.001*
Corpus gastritis (*n* = 311)	116 (37.3%)	< 0.001*
Pangastritis (*n* = 229)	106 (46.3%)	< 0.001*
Gastric ulcer (*n* = 28)	5 (17.9%)	0.333*
Duodenal ulcer (*n* = 48)	29 (60.4%)	< 0.001**

*Pearson chi-square test

**Yates’ correction test

☨*p* values were calculated by comparing *H. pylori* positivity between patients with and without the respective endoscopic finding

The prevalence of *H. pylori* increased with the degree of histological severity of active and chronic gastritis (Table 4), and severe gastritis was associated with higher bacterial density in biopsy samples (*p* < 0.001). In this cohort, premalignant lesions were uncommon; gastric atrophy was detected in 2.3% of patients (*n* = 30/1300), most of which were grade 1 (*n* = 24), while 6 patients had grade 2 atrophy and no patients had grade 3 atrophy. Intestinal metaplasia was identified in 1.38% (*n* = 18), predominantly grade 1 (*n* = 15), one patient with grade 2 and two patients with grade 3. Children with glandular atrophy or intestinal metaplasia had nearly twice the prevalence of *H. pylori* infection compared to those without these lesions (Table 4).

**Table 4:** Association of histopathological findings with *H. pylori* infection

Histopathological findings		*H. pylori* (+)	*p*☨
Antrum chronic inflammation	Mild *n* = 434	48 (11.1%)	< 0.001*
	Moderate–severe *n* = 347	283 (81.6%)	
Antrum active inflammation	Mild *n* = 237	192 (81%)	< 0.001*
	Moderate–severe *n* = 111	107 (96.4%)	
Corpus chronic inflammation	Mild *n* = 444	161 (36.3%)	< 0.001*
	Moderate–severe *n* = 191	164 (85.9%)	
Corpus active inflammation	Mild *n* = 207	189 (91.3%)	< 0.001*
	Moderate–severe *n* = 56	53 (94.6%)	
Gastric glandular atrophy (*n* = 30)		14 (46.7%)	0.015**
Gastric intestinal metaplasia (*n* = 18)		9 (50%)	0.027***

*Linear-by-linear association

**Yates’ correction test

***Fisher’s exact test

☨*p* values were calculated by comparing *H. pylori* positivity between patients with and without the respective histological finding

In multivariate logistic regression analysis, older age, lower height Z-score, dyspeptic symptoms, and refractory IDA were independently associated with *H. pylori* infection. Among endoscopic findings, antral nodularity, pangastritis, and duodenal ulcer were also independently associated with infection. Histopathologically, chronic inflammation in both the antrum and corpus was independently associated with *H. pylori*, whereas glandular atrophy and intestinal metaplasia were not independently associated (Table 5).

**Table 5:** Multivariate logistic regression models evaluating clinical-demographic, endoscopic, and histopathological factors associated with *H. pylori* infection

Clinical and demographic variables		OR	95% CI	*p*
Age	Per year increase	1.171	1.135–1.208	** < 0.001**
Height Z-score	Per 1 SD increase	0.868	0.792–0.950	**0.002**
Gender	Female	Ref		
	Male	1.068	0.817–1.398	0.629
Dyspepsia	Absent	Ref		
	Present	1.588	1.208–2.089	**0.001**
Refractory IDA	Absent	Ref		
	Present	1.696	1.029–2.796	**0.038**
Endoscopic variables				
Antral nodularity	Absent	Ref		
	Present	36.077	25.347–51.349	** < 0.001**
Pangastritis	Absent	Ref		
	Present	1.971	1.295–3.000	**0.002**
Duodenal ulcer	Absent	Ref		
	Present	2.682	1.175–6.122	**0.019**
Histopathologic variables				
Antrum chronic inflammation	Absent	Ref		
	Present	17.381	6.187–48.828	** < 0.001**
Corpus chronic inflammation	Absent	Ref		
	Present	24.588	12.647–47.804	** < 0.001**
Glandular atrophy	Absent	Ref		
	Present	0.963	0.411–2.259	0.931
Intestinal metaplasia	Absent	Ref		
	Present	1.231	0.410–3.697	0.711

*OR* odds ratio, *Ref* reference category, *CI* confidence interval; ORs adjusted within each model

## Discussion

In this study, we demonstrated a decline in the prevalence of *H. pylori* infection in children undergoing UGE throughout the study period. *H. pylori* positivity increased with age and was associated with impaired growth, dyspeptic symptoms, refractory IDA, characteristic endoscopic findings such as antral nodularity, and histological gastritis.

*H. pylori* infection remains one of the most common infections worldwide; however, recent studies have reported a decline in prevalence, largely attributed to improved sanitation and eradication strategies in adults [15, 16]. Despite this trend, its global distribution remains highly heterogeneous across regions and socioeconomic settings [7, 8]. Differences in reported prevalence can be partly explained by variations in diagnostic modalities such as stool antigen testing, urea breath testing, and endoscopy-based methods; these differences should be considered when comparing studies. Among available diagnostic methods, bacterial culture constitutes a valuable diagnostic test for *H. pylori* infection. However, it is technically demanding, requires specialized transport conditions and laboratory expertise, and was not routinely performed at our center during the study period. Instead, *H. pylori* infection was diagnosed using histopathology in combination with a positive RUT in accordance with the 2016 ESPGHAN/NASPGHAN guidelines [11]. This diagnostic strategy remains consistent with the confirmation criteria outlined in the updated 2024 recommendations [17].

Our study was conducted in a tertiary-care center using an endoscopy-based diagnosis in symptomatic children; therefore, the cohort reflects a specific clinical group rather than the general pediatric population. In endoscopy-based studies of symptomatic children, *H. pylori* prevalence has varied across regions, reported as 37.3% in Portugal and lower rates of 16.5% and 12.8% in Lebanon and China, respectively [18–20]. In our cohort, prevalence was 25.8%, falling between these estimates. Despite similar endoscopic settings, direct comparisons remain challenging because differences in socioeconomic factors, access to healthcare, referral patterns, and institutional practices can influence observed rates.

Recent meta-analyses have reported a global pediatric *H. pylori* prevalence of approximately 30–35%, with inconsistent evidence regarding temporal trends, as some studies observed report rates while others suggest a decline [6, 10]. However, endoscopy-based studies focusing on symptomatic children have demonstrated declining prevalence over time in several countries, including China, Poland, Brazil, and Turkey [21–24]. In line with these reports, we observed a marked decrease in *H. pylori* prevalence from 47.9% in 2010 to 23.4% in 2020 among symptomatic children undergoing endoscopy. The decline was significant in school-aged children and adolescents but not in preschool children, likely reflecting the already low baseline prevalence in younger age groups [10]. Improvements in living standards, sanitation, and socioeconomic conditions may have contributed to this decline. Reduced household crowding, lower birth rates, and successful eradication therapies in adults may have decreased intra-familial transmission [10, 25–29]. In our country, the crude birth rate was 20.3 per thousand in 2001 and declined to 13.3 per thousand by 2020 [30], while per capita national income increased from 3583 to 8638 USD during the same period [31]. These changes may have contributed to the reduced frequency of endoscopically detected *H. pylori* infection among symptomatic children in our country. Increased public awareness of *H. pylori* transmission may also have played a role in this decrease.

*H. pylori* infection is typically acquired early in life and increases with age [2, 10]. In our cohort, as reported previously, older age was independently associated with *H. pylori* infection. No significant association was observed between gender and *H. pylori* infection in our study, suggesting that gender is not a consistent risk factor for infection [8, 10, 32–34]. Our retrospective study did not include data on *H. pylori* infection in family members, and no association was found between family history of gastrointestinal disease and *H. pylori* positivity. Previous studies have reported conflicting results [34, 35].

Although current pediatric guidelines suggest that dyspeptic symptoms other than peptic ulcer disease are not strongly associated with *H. pylori* infection [17], dyspepsia remained independently associated with *H. pylori* in our cohort. This discrepancy may reflect referral bias, as tertiary-care centers evaluate a higher proportion of patients with persistent or treatment-resistant gastrointestinal symptoms. We did not observe any association between *H. pylori* and gastroesophageal reflux symptoms, endoscopic esophagitis, and hematemesis and/or melena, consistent with previous pediatric studies [36–39].

*H. pylori* infection may adversely affect appetite, dietary intake, and growth. Studies have reported reduced leptin, ghrelin, and IGF-1 levels in infected children, with improvement in growth parameters after eradication therapy [28, 40, 41]. Recent meta-analyses suggest a negative effect on linear growth [42, 43], although other studies have found no significant association with anthropometry [44–46]. Because both growth and *H. pylori* infection are influenced by socioeconomic and cultural factors, establishing a causal relationship remains difficult [28, 47, 48]. In our cohort, *H. pylori*–positive children had significantly lower height and weight Z-scores, with low height Z-score remaining independently associated with infection, consistent with recent meta-analyses [42, 43]. Nevertheless, we found no difference in *H. pylori* prevalence between children with and without growth faltering/failure, indicating that the relationship between *H. pylori* infection and growth should be interpreted cautiously and does not necessarily imply causality.

*H. pylori* infection has been proposed to contribute to IDA through impaired iron absorption, gastrointestinal blood loss, and bacterial competition for iron [49]. Meta-analyses suggest an association with reduced iron stores in children, although individual studies have reported conflicting results [50–53]. In our cohort, no overall association was observed between anemia and *H. pylori* infection; however, refractory IDA remained independently associated with infection, supporting guideline recommendations to evaluate *H. pylori* in unexplained anemia after exclusion of nutritional causes and bleeding [17].

Consistent with previous reports [54, 55], *H. pylori* was found to be more frequent in patients with antral nodularity and gastritis. Duodenal ulcers, but not gastric ulcers, were also associated with infection, reflecting the increasing contribution of non–*H. pylori* causes to gastric ulcer etiology as *H. pylori* prevalence declines [56]. In multivariate analysis, antral nodularity, pangastritis, and duodenal ulcer remained independently associated with *H. pylori* infection, with antral nodularity showing the strongest association. In contrast, *H. pylori* infection was lower in patients with antral gastritis without nodularity in our cohort, suggesting that endoscopic gastritis without nodularity may not represent the typical endoscopic phenotype of infection in pediatric population. *H. pylori* infection was detected in 7.2% of patients with endoscopically normal gastric mucosa, underscoring the importance of taking biopsies during UGE in children.

Histological evaluation in our study demonstrated a strong association between *H. pylori* infection and active and chronic inflammation in both the antrum and corpus. Inflammation severity correlated with bacterial density, supporting the role of *H. pylori* in mucosal injury [57, 58]. Glandular atrophy and intestinal metaplasia, rare in childhood and typically associated with *H. pylori*, were observed at rates comparable to previous studies from Turkey [59] but lower than those reported from some Asian populations [60]. Given the pediatric population, mild (grade 1) atrophic changes may reflect inflammatory or edema-related alterations rather than established glandular loss, and alternative etiologies such as autoimmune gastritis should also be considered. In univariate analysis, *H. pylori* infection was more frequent among patients with glandular atrophy and intestinal metaplasia; however, these associations were not seen in multivariate analysis. This suggests that such histopathological changes reflect chronic infection rather than independent predictors of *H. pylori*. As these precancerous changes regress after eradication, their detection and management in childhood remain clinically important.

In conclusion, we demonstrated a significant decline in *H. pylori* prevalence among children undergoing endoscopy between 2010 and 2020. This downward trend may reflect improvements in socioeconomic status, sanitation, school-based education, and the effective eradication of *H. pylori* in adults. Childhood *H. pylori* infection has been associated with growth impairment, refractory IDA, dyspepsia, gastritis, duodenal ulcer, and precancerous lesions such as glandular atrophy and intestinal metaplasia. Given the long-term clinical consequences, accurate diagnosis, effective treatment, and preventive measures such as education about transmission routes and improved hygiene are essential to reduce prevalence to the levels seen in developed countries.

## Data Availability

No datasets were generated or analysed during the current study.

## Abbreviations

BMI: Body mass index
CIs: Confidence intervals
ESPGHAN: European Society for Paediatric Gastroenterology, Hepatology and Nutrition
*H. pylori*: *Helicobacter pylori*
IQR: Interquartile range
IDA: Iron deficiency anemia
NASPGHAN: North American Society for Pediatric Gastroenterology, Hepatology and Nutrition
ORs: Odds ratios
RUT: Rapid urease testing
SD: Standard deviations
SPSS: Statistical analyses were performed using the Statistical Package for the Social Sciences
UGE: Upper gastrointestinal endoscopy
